# Behind the Scenes at *Investigative Ophthalmology & Visual Science*

**DOI:** 10.1167/iovs.66.2.19

**Published:** 2025-02-06

**Authors:** Joseph Carroll

**Affiliations:** *Editor-in-Chief*; 1 Department of Ophthalmology & Visual Sciences, Medical College of Wisconsin, Wisconsin, United States

Having recently completed my second year of service as Editor-in-Chief (EIC) at *Investigative Ophthalmology & Visual Science* (*IOVS*), I wanted to take the opportunity to communicate about the status of our journal—both to recognize some changes that have taken place and to identify areas where we are working to improve.

## Current Journal Statistics

**Figure 1.**
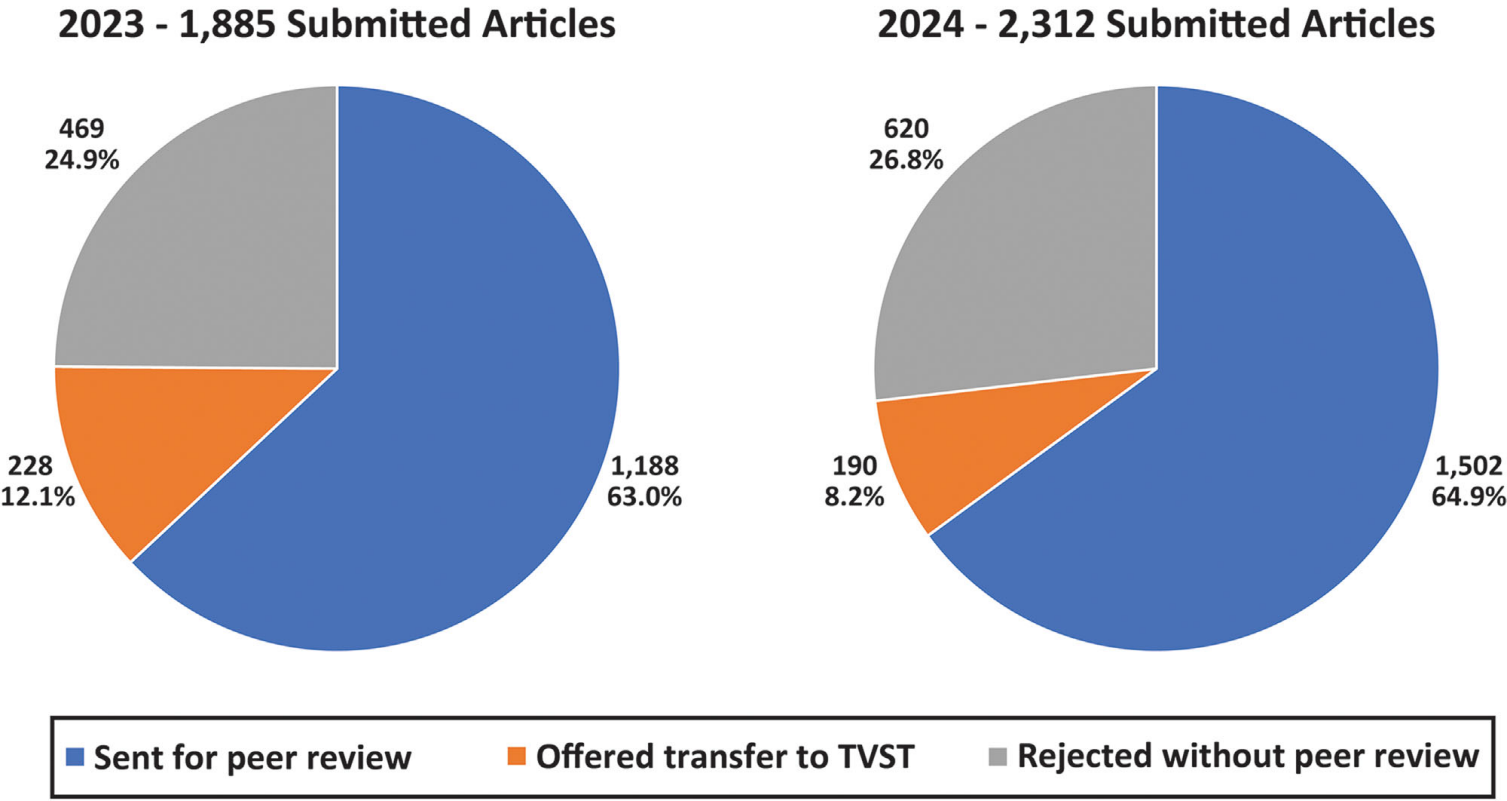
Initial action on manuscripts submitted to *IOVS* in 2023 and 2024. These numbers exclude Errata, Letters to the Editor, and Author Responses to Letters. There were 24 such submissions in 2023 and 34 in 2024.

*IOVS* aims to be a top choice for authors looking to publish impactful and statistically valid studies in all areas of the visual system. Accordingly, *IOVS* continues to be a popular destination for authors. Shown in Figure 1 is a summary of the submissions in each of the past two years—we saw an increase of 427 articles from 2023–2024 (a 23% increase)! One goal has been to reduce the number of papers offered transfer to *Translational Vision Science & Technology* (*TVST*), and we improved somewhat, going from nearly 12% of submitted articles being offered transfer to just over 8%. This is due largely to authors more carefully reviewing the author guidance on *IOVS* versus *TVST* scope (see below), and the hope is to further reduce transfer offers in 2025. Authors often ask why articles are rejected without peer review—with so many submissions we need to have some initial filtering of manuscripts—decisions to not send papers for review is sometimes made by me as EIC, but also frequently with input from an Associate Editor (AE) and/or Editorial Board Member (EBM). Some examples include plagiarism, overall low-quality presentation, a lack of novelty or rigor, and being outside the scope of *IOVS*. Having been on the receiving end of many desk rejections myself, I realize this is generally not welcome news for an author. My goal is to provide these decisions quickly, to provide authors with the freedom to resubmit their manuscript elsewhere without delay. However, our growth in submissions has been comprised largely of high-quality manuscripts. As can be seen in Figure 1, of the 427 additional submissions from 2023–2024, about 74% (314 submissions) were sent for peer review, meaning that there is an increased need for high-quality peer review, which I touch on toward the end of this editorial.

## By the Numbers: Peer Review at *IOVS*

As I had mentioned when I was running for the position of EIC, one of my main areas of focus is the author experience—specifically, time to first decision on submitted manuscripts. At *IOVS*, peer-reviewed manuscripts go through several stages on their way to a first decision, including assessments by the EIC, AE, EBM, and peer reviewers. To identify opportunities for improvement, I undertook an in-depth analysis of all submitted manuscripts that were sent for peer review. The goal was to conduct an accurate process mapping to identify sources of “waste” in the *IOVS* peer review process. By waste, I am referring to anything that does not add value to the process—in this case, anything that does not improve either the quality and/or timeliness of the review. Table 1 provides high-level data for the past four years. The mean time to first decision increased from 38.12 days for 2021 to 40.93 days in 2024 (median time increased from 35 to 38 days). Over this same time, we saw a 26% increase in the number of submitted articles (1831 in 2021 and 2312 in 2024). The additional influx of articles has certainly created burden on our peer review system and makes it challenging to improve efficiency.

**Table 1.** Peer Review Statistics at *IOVS* (2021–2024)

		Days to First Decision		Days to Return Review	
Year	Number of Submissions	Mean	Median	Mean	Median
2021	1831	35	38.12	16.00	15
2022	1833	38	41.88	16.35	15
2023	1885	39	44.12	16.14	15
2024	2312	38	40.93	15.84	15
AVG	1965	37.5	41.26	16.08	15

Despite this burden, the time a given article spends with peer reviewers hasn't changed much in the past four years. As shown in Table 1, the average time it takes for a reviewer to return their review after accepting the invite is just over two weeks (which is the turnaround time we request). This time is considered “value added” time in our process mapping. When looking at the overall journey from manuscript submission to first decision over the past four years, we find that almost 17% of the time to first decision comes *prior* to the first reviewer ever being assigned. This points us to an opportunity for improvement without compromising peer-review quality. Much of this delay is from EBMs not responding to invitations, AEs being delayed in inviting EBMs, or a combination of the two. We continue to work on decreasing this initial delay. Once reviews are in, another ∼15% of first decision time can be attributed to the EBM and AE decision process. This should not be considered waste, however, because there is often necessary adjudication or deliberation in cases of disparate reviews.

One area we have seen an increase is in the number of reviewers who don't respond to an invitation to review (Table 2). In 2024, about one-third (*n* = 2,335) of the *IOVS* review invitations went unanswered. These instances add significant time to the overall time to first decision based on our current invitation system (eJP)—the system will wait a couple days for a response then send the reviewer a reminder, then wait a couple more days before declaring the reviewer a “no response” and moving to invite the next reviewer. As such, a single reviewer not responding to their invite can add up to one week to the first-decision time for an article (because reminders are generally only sent on business days). There have been some extreme outliers that can taint individual author experiences (and skew the journal statistics)—one article had 34 reviewer invitations! Of these invited reviewers, 15 did not respond and 16 declined. The three reviewers who reviewed the paper took an average of 19 days to do their review (which is very reasonable given the requested two-week turnaround). Unfortunately, this is not an exception—almost 20% of our manuscripts in 2024 had more than 40% of their reviewer invites go without a response, and 8% of our 2024 manuscripts had half of their reviewer invites go unanswered. Overall, there is a significant positive correlation between the number of invitations that were not responded to and the total days to first decision (*r* = 0.45, *P* < 0.0001). Although cleaning up our reviewer database to ensure invitations are going to currently active people at valid email addresses will help reduce some of this, my request is that if invited to review (whether it be by *IOVS* or another journal), you respond (even if you must decline). If you do decline, we very much appreciate suggestions for reviewers and have made it easier for EBMs to act on these suggestions.

**Table 2.** *IOVS* Reviewer Invitations (2021–2024)

	Total Invited		Accepted		Declined		No-Response	
Year	Total	AVG*	Total	AVG*	Total	AVG*	Total	AVG*
2021	4511	6.44	1435	2.05	2146	3.06	930	1.33
2022	4360	6.67	1349	2.06	1989	3.04	1022	1.56
2023	6390	6.85	1951	2.09	2436	2.61	2003	2.15
2024	7402	6.78	2338	2.14	2729	2.50	2335	2.14
AVG	5666	6.69	1768	2.09	2325	2.80	1572	1.80

* Average number per reviewed manuscript.

## What's New at *IOVS*?

On the outside, *IOVS* looks pretty much the same since I began my five-year term as EIC: same website, same logo, and same great science. However, to conclude that the journal has been idle over the past two years would be grossly inaccurate. Many changes have been implemented to improve the overall author experience, although I believe that many will spill over and positively impact our reviewers and editors as well. It is with this in mind that we've undertaken a series of tweaks/changes over the past 18–24 months.

**Figure 2.**
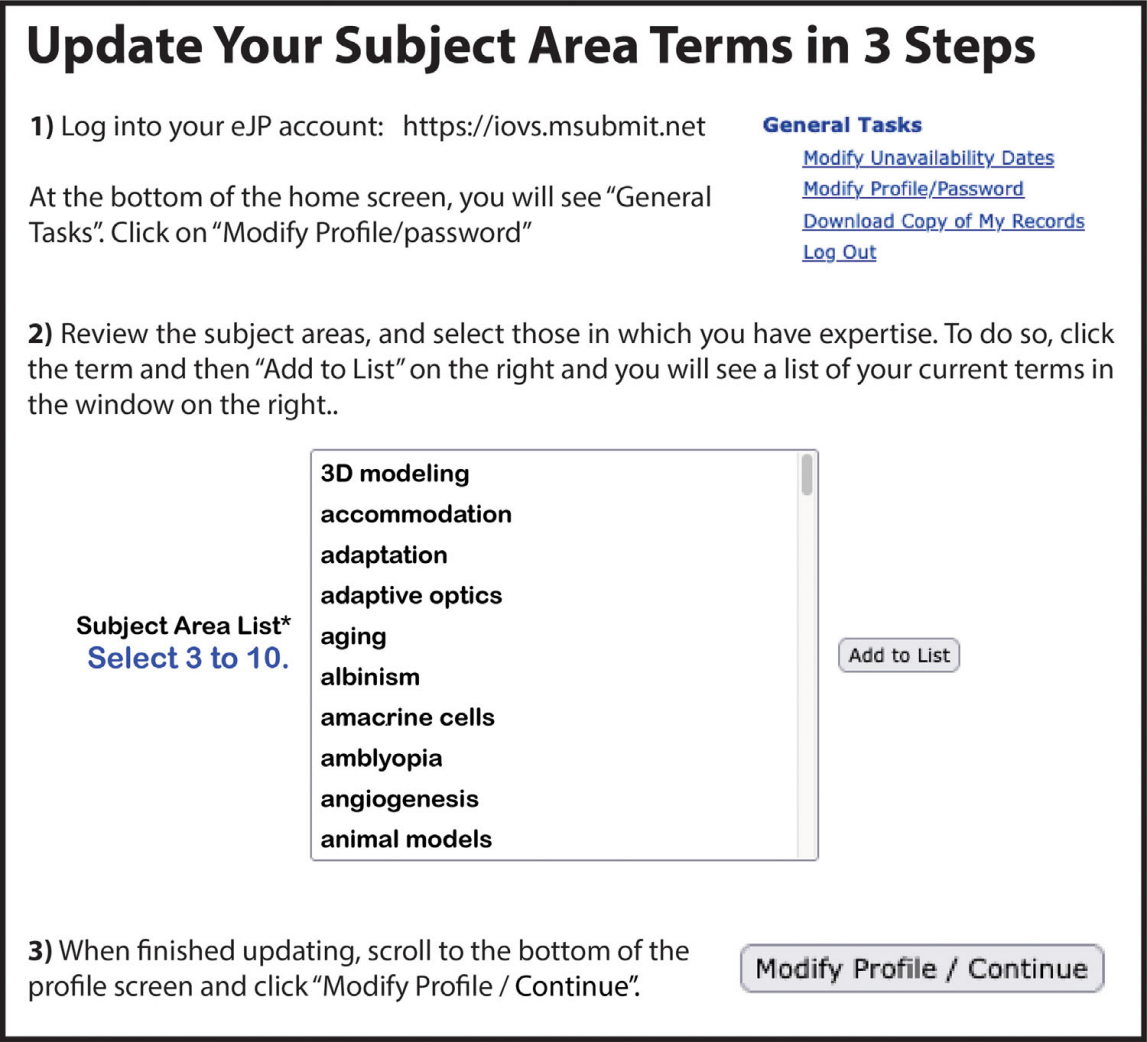
How to update your eJP user profile.

Perhaps the biggest change was the merging of the *IOVS*, *TVST*, and *Journal of* *Vision* databases. This means that instead of having a separate user profile for each journal, you have just one. This allows AEs and EBMs across the three journals to share reviewer statistics, which in theory could reduce burden for individual reviewers who may have previously received competing invites from the different ARVO journals. This experiment has largely been successful, although there have been some growing pains as we realize many authors and reviewers have not updated their profiles in quite some time. Why does having a current profile matter? Well, it reduces bounced emails and helps editors identify appropriate reviewers with the right scientific expertise for a given article. Speaking of expertise, we also revised and condensed the cross-journal subject area list used by authors to tag their manuscripts and by reviewers in their profiles. This was over 500 terms and meant that the reviewer database would often not effectively match candidate reviewers to a submitted article because of minor differences in subject area words. For example, we actually had separate subject areas for *electroretinogram* and *electroretinography*—meaning if you selected one term for your paper and someone else had the other term as their listed expertise, the system would not identify them as a good match to review the paper! We have merged and culled the subject area terms list by more than half. However, the positive impact of this effort requires authors and reviewers to login and update their profile to select their expertise from the new terms list. We are exploring automated ways to update subject area terms based on characteristics of manuscripts a given user publishes and/or reviews, but in the meantime please see Figure 2 for guidance on how to update *your* profile! Please note that your account as an author and as a reviewer is the same, so that anyone in our database who has been an author may be found when our editors search for reviewers, and anyone who has been a reviewer can be found and added as a co-author during the manuscript submission process (see below). In both cases an up-to-date profile is essential.

**Figure 3.**
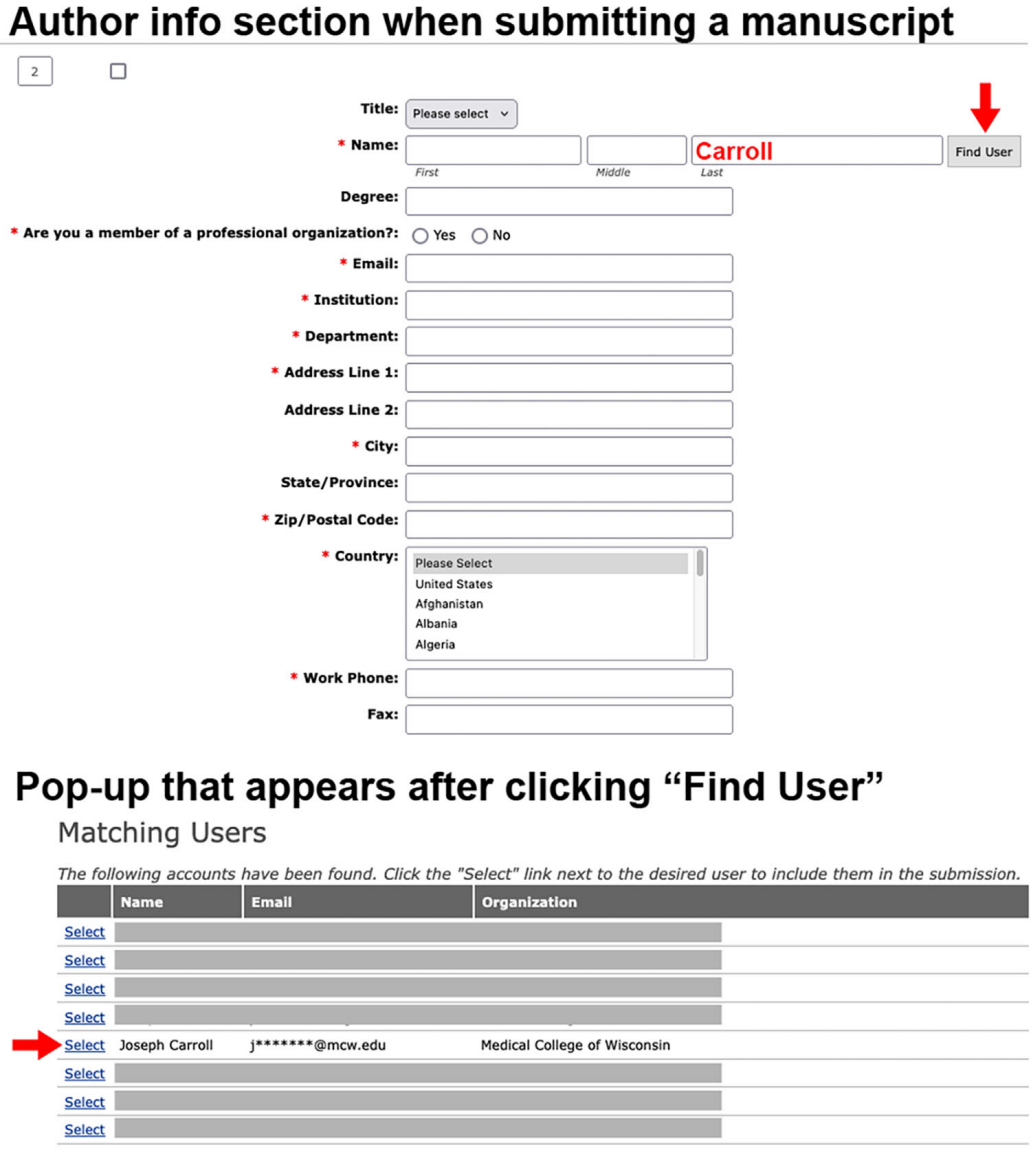
Adding co-author information automatically.

Another change related to our database has to do with manuscript submission portal used by authors. When submitting a manuscript, authors are required to input the details of their co-authors. Previously, this information would have to be entered manually for each manuscript. This resulted in the creation of thousands of duplicate profiles in our database—if the information entered didn't match an existing profile identically, a new one would be created (even if the mismatch was something trivial, like a spelling of the department affiliation). However, after doing an internal audit, I discovered that there was a feature that had not been activated to our live submission site. Figure 3 details the “Find User” function. Basically, for each co-author you want to add during submission, simply type in their last name and click on “Find User.” The system will then show you a list of existing profiles that match, and you can select the one you want to pull in. This reduces effort for the submitting author and nearly eliminates the accidental generation of duplicate accounts for the same user. We are exploring additional automated ways to extract author information, but for now, we are hoping authors will use this “Find User” feature rather than manually type in co-author information during the submission process.

**Figure 4.**
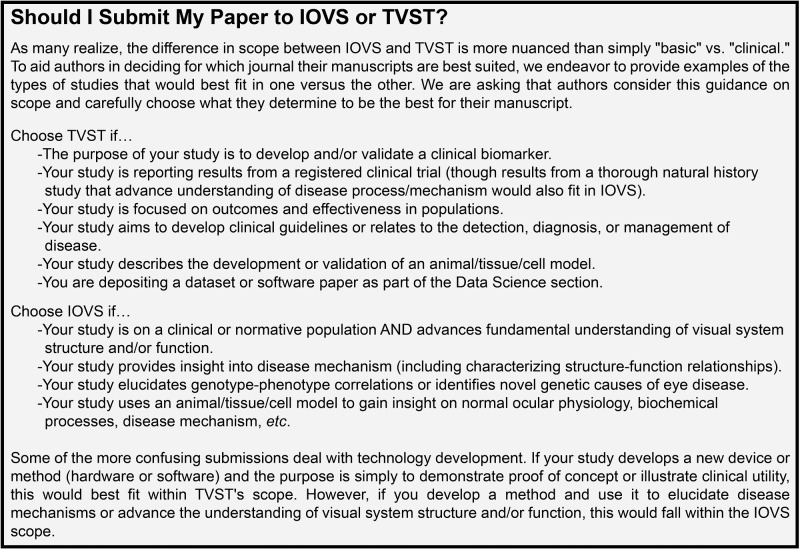
Current scope considerations for *TVST* and *IOVS*.

We've also sought to provide better guidance to authors. If you have not done so recently, I encourage you to review the *IOVS* Instructions to Authors (https://iovs.arvojournals.org/ss/forauthors.aspx). Here you will see that *IOVS* has recently adopted the ARVO Best Practices for Eye Tissue, which we think will positively impact the rigor and reproducibility of published studies. Together with the *TVST* EIC (Dr. Roy Chuck), we crafted revised guidance to help authors select the best journal for their study, as there had previously been some uncertainty as to the overlap between the journals. Although the guidance is far from perfect, we hope it helps authors choose the most appropriate journal (Fig. 4). We also crafted new guidance on the use of AI in articles, which aligns in large part with policies set forth by other journals and the NIH. An important point is that currently we do not allow editors or reviewers to use AI tools to evaluate manuscripts. *IOVS* is looking into services (similar to our plagiarism-detection process through iThenicate) to conduct such reviews in a closed system to protect the confidentiality of the peer review system. Finally, we have a working group drafting guidance on genetics studies—including Mendelian randomization studies and depositing of genetic data. We hope to have this genetic guidance available to authors later in 2025. If you have additional suggestions for improvements to our Instructions to Authors, please email me.

Speaking of email, one of my favorite changes involved the creation of a new ARVO-owned EIC email (iovs.eic@arvo.org). Rather than use an institutional email that changes with each new EIC, it seemed wise to adopt a single email to house all *IOVS* EIC communications. This should especially help during the transition between EICs that occurs every five years to ensure that communication regarding specific manuscripts doesn't slip through the cracks. Additionally, this addresses confidentiality issues—many university-owned emails are not private. Institutional technology departments can review emails, and email at U.S. public institutions cannot be considered confidential because of Freedom of Information Act policies. As such, managing confidential peer-review processes for *IOVS* seems more appropriate using an ARVO-managed email system and I am happy to have spearheaded this change.

Finally, I have undertaken efforts to expand the editorial board—both in size and diversity (see https://iovs.arvojournals.org/ss/editorial_board.aspx for the current Editorial Board roster). To reduce overall workload, I increased the number of AEs from nine when I started to 19 currently. About 37% of the AEs are based outside the United States (five different countries), and 42% are female. Among the 125 EBMs, about 49% are female and 38% are based outside the United States (20 different countries). Over the next three years I will continue to expand the Editorial Board as needed, with a focus on further diversification.

## Moving Forward

I am excited about the next few years for *IOVS*. I plan to expand our Reviewer-in-Training program, which I hope will grow the database of available reviewers. In addition, I anticipate we will continue to participate in the Council for Vision Editors Fellowship program, which is a cross-journal collaboration to help train the next generation of reviewers and editors.^1^ We are also introducing new reviewer and editorial awards at ARVO 2025, something that is long overdue! Finally, we have already begun the process of launching the election plan for the next EIC—so watch for more communication about this later this spring.
